# Land use effects on soil microbiome composition and traits with consequences for soil carbon cycling

**DOI:** 10.1093/ismeco/ycae116

**Published:** 2024-10-07

**Authors:** Lisa Cole, Tim Goodall, Nico Jehmlich, Robert I Griffiths, Gerd Gleixner, Cecile Gubry-Rangin, Ashish A Malik

**Affiliations:** School of Biological Sciences, University of Aberdeen, St Machar Drive, Aberdeen AB24 3UU, United Kingdom; UK Centre for Ecology and Hydrology, Benson Lane, Crowmarsh Gifford, Wallingford OX10 8BB, United Kingdom; Department of Molecular Systems Biology, Helmholtz Centre for Environmental Research-UFZ, Permoserstrasse 15, Leipzig 04318, Germany; UK Centre for Ecology and Hydrology, Benson Lane, Crowmarsh Gifford, Wallingford OX10 8BB, United Kingdom; School of Natural Sciences, Bangor University, Deiniol Road, Bangor LL57 2UR, United Kingdom; Max Planck Institute for Biogeochemistry, Hans-Knoell-Strasse 10, Jena 07745, Germany; School of Biological Sciences, University of Aberdeen, St Machar Drive, Aberdeen AB24 3UU, United Kingdom; School of Biological Sciences, University of Aberdeen, St Machar Drive, Aberdeen AB24 3UU, United Kingdom; UK Centre for Ecology and Hydrology, Benson Lane, Crowmarsh Gifford, Wallingford OX10 8BB, United Kingdom; School of GeoSciences, University of Edinburgh, The King’s Buildings, Alexander Crum Brown Road, Edinburgh EH9 3FF, United Kingdom

**Keywords:** soil microbiome, carbon cycling, land use intensity, soil pH, metaproteomics, metabarcoding, soil organic carbon, 13C labeling, carbon use efficiency, soil health

## Abstract

The soil microbiome determines the fate of plant-fixed carbon. The shifts in soil properties caused by land use change leads to modifications in microbiome function, resulting in either loss or gain of soil organic carbon (SOC). Soil pH is the primary factor regulating microbiome characteristics leading to distinct pathways of microbial carbon cycling, but the underlying mechanisms remain understudied. Here, the taxa-trait relationships behind the variable fate of SOC were investigated using metaproteomics, metabarcoding, and a ^13^C-labeled litter decomposition experiment across two temperate sites with differing soil pH each with a paired land use intensity contrast. ^13^C incorporation into microbial biomass increased with land use intensification in low-pH soil but decreased in high-pH soil, with potential impact on carbon use efficiency in opposing directions. Reduction in biosynthesis traits was due to increased abundance of proteins linked to resource acquisition and stress tolerance. These trait trade-offs were underpinned by land use intensification-induced changes in dominant taxa with distinct traits. We observed divergent pH-controlled pathways of SOC cycling. In low-pH soil, land use intensification alleviates microbial abiotic stress resulting in increased biomass production but promotes decomposition and SOC loss. In contrast, in high-pH soil, land use intensification increases microbial physiological constraints and decreases biomass production, leading to reduced necromass build-up and SOC stabilization. We demonstrate how microbial biomass production and respiration dynamics and therefore carbon use efficiency can be decoupled from SOC highlighting the need for its careful consideration in managing SOC storage for soil health and climate change mitigation.

## Introduction

Soils are under pressure to deliver multiple ecosystem services, especially food production. This has led to the expansion of agriculture into pristine environments and increased land use intensification. There is a growing recognition that the intensive use of soils is detrimental to soil health, changing soils’ inherent biodiversity and risking the services that they provide [1, 2]. The world’s soils have historically lost 133 Pg of carbon due to land use intensification [3]. However, degraded soils low in organic matter also represent an opportunity to adopt regenerative management promoting soil carbon storage that may help mitigate this issue [1, 4, 5]. To better achieve this aim, it is vital to understand the role of soil microbes in carbon cycling, as the microbiome plays an important role in soil-atmosphere carbon exchange by determining the fate of carbon in soils [6].

A new paradigm recognizes the direct, significant contribution of microbes in transforming photosynthetically-derived carbon into soil organic carbon (SOC) [7], by stabilizing dead microbial biomass (necromass) onto mineral surfaces to enable persistent, long-term carbon storage [8]. Microbial CUE is a vital ecosystem trait that determines soils’ ability to accumulate carbon [9] and is measured as the incorporation of organic carbon from the environment into microbial biomass through growth [10, 11]. A higher microbial CUE implies more efficient biomass production and a lower respiratory loss [12]. Increased growth and turnover of microbes results in a bigger necromass pool. Biomass turnover can be caused by a variety of process such as microbivory, predation, natural cell death and viral lysis. Necromass that is stabilized on association with mineral surfaces can form persistent SOC thereby promoting soil carbon storage [13]. As microbes become more efficient in using carbon, higher carbon storage is observed in soils, a pattern that has been demonstrated at the global scale using meta-analysis and modeling approaches [14]. Increased microbial CUE therefore offers the potential to increase the necromass pool for stabilization in the mineral-associated organic matter (MAOM) resulting in potential long term SOC storage.

Microbiome diversity and function are responsive to environmental gradients [15–17], and microbial biomass is generally greatest under lower intensity land use [18, 19]. Given the degraded state of many of the world’s agricultural soils that have lost SOC, croplands represent a habitat where carbon storage could be promoted through microbiome-mediated processes [20]. Therefore, it is crucial to understand how land use intensification impacts key microbial traits such as CUE [21–23]. This knowledge would enable us to better manage degraded grassland soils to enhance microbial CUE and promote SOC stabilization, providing many benefits for soil health, soil biodiversity, and climate change mitigation [4, 24, 25].

A positive relationship between microbial biomass and SOC concentration has been observed across 21 paired land use contrasts in the UK [26]. However, land use intensification effects on community-level CUE were complex and were better explained through interactions of multiple soil properties. Of these, soil pH was identified as the dominant factor, as converting grasslands to cropland tends to increase soil pH and microbes are sensitive to pH [26]. Soil pH has been previously found to be the main factor influencing soil microbial diversity [27, 28]. The UK-wide study suggested two distinct, pH-dependent mechanisms of soil carbon accumulation [26]. Acid, wet, and anoxic conditions limit microbial growth and decomposition [26], accumulating part-decomposed plant material at the soil surface resulting in high SOC in upper soil horizons [29]. In contrast, well-drained neutral to alkaline pH soils provide conditions more conducive to microbial growth, promoting necromass generation for stabilization as SOC [26]. Thus, soil pH can be used as a proxy to study the divergent effect of land use intensification on soil microbiomes and carbon cycling.

The trait-based life history strategies of the resident microbiome can explain the divergent mechanisms of microbial SOC accumulation. A life history framework has been proposed for microbes classifying them into three main strategies: high yield (Y), resource acquisition (A) and stress tolerance (S) with multiple underlying traits [30]. These traits correlate due to physiological or evolutionary trade-offs, influenced by the environmental conditions such as resource availability and abiotic stress [26, 30]. In low resource environments, typical of high land use intensity soils (e.g. arable systems where plant biomass inputs to soil tend to be low), traits that enable microbial survival and activity include investment into the production of extracellular enzymes for resource acquisition pathways [30, 31]. In temperate soils under conversion from grassland to cropland, microbes are exposed to increased frequency of drought stress as tillage leads to soil aggregate disruption and lower water holding capacity [32]. Investment in stress tolerance in high land use intensity soils can often be observed with chaperone proteins such as Chaperonin GroEl, which prevent stress-induced misfolding of proteins [26]. These increased cellular investments into stress alleviation and resource acquisition trade off with microbial growth yield due to the diversion of resources from growth and biosynthesis. The reduced biomass (and subsequent necromass pool) and the increased respiratory loss reflect lower potential SOC accumulation rates [26, 30]. Furthermore, under intense abiotic stress, such as drought, microbes might also shift to a dormancy state, reducing microbial CUE [33, 34].

While microbial community-level traits, such as CUE, have been linked to ecosystem measures, such as changes in SOC, identifying taxonomic groups contributing to higher CUE is challenging. Previous studies have aimed to do this, by assigning microbial taxa to trophic groups or life history strategies, such as the copiotroph–oligotroph dichotomy [35, 36]. It was observed that copiotrophs invest in a competitive strategy and have a high maintenance respiration, which reduces their CUE. In contrast, oligotrophs maintain growth over respiration in low quality resource environments, thereby increasing their CUE [36, 37]. However, the copiotroph–oligotroph dichotomy does not exist at broader levels of taxonomic linages [38]. Therefore, linking a comprehensive set of traits (such as those for Y-A-S life history strategies) to taxonomic identity is essential to better understand how organismal physiology influences ecosystem-level processes.

This study investigated the microbial community response to land use intensification by comparing local over-the-fence land use contrasts in two temperate sites of low- and high-soil pH. Comparing adjacent land use intensity treatments allowed us to study the effect of land use intensification while the climate and parent material remained constant. The aim was to understand how taxonomic and trait shifts with land use intensification impact soil carbon cycling. Our current understanding of the microbial traits underpinning SOC stabilization processes is mainly obtained through analyzing a community response, often using an emergent trait such as CUE. In addition to this approach, we aimed to identify how changes in the abundance of dominant microbial taxa caused by land use intensification led to shifts in key microbial traits, emergent ecosystem CUE [9], and SOC decomposition and stabilization rates [26]. We hypothesize that increased land use intensification impacts soil properties, with a shift in the microbial community from high growth yield taxa to resource acquiring and stress tolerant taxa, resulting in lower CUE and SOC stabilization. Using metaproteomics and metabarcoding, we identified the dominant taxonomic groups with different Y-A-S traits and related them to ecosystem CUE estimates. Therefore, this study demonstrates how land use intensification selects microbial communities with variable organismal traits impacting soil carbon cycling.

## Materials and methods

### Site description

To understand how microbial taxonomy and traits influence soil carbon dynamics in soils of differing land use intensity, we chose two sites with contrasting pH that were previously studied as part of a landscape scale survey [26]. Each site had two locally adjacent over-the-fence land use contrasts of low and high land use intensity. Low-pH site in Kirkton, Perthshire, UK: at this site, the low land use intensity treatment consisted of historically undisturbed soils (pH 5.2) representative of wet acid upland podzols with high SOC in the upper horizons. It was a poor semi-improved grassland with no history of cultivation and was grazed. The contrasting high land use intensity treatment consisted of soils improved to support agricultural activities by drainage and liming, this raised soil pH to 6.4. It was fertilized and supported a re-seeded grassland and winter wheat rotation. High-pH site in Parsonage Down, Wiltshire, UK: at this site, the low land use intensity treatment consisted of undisturbed soils that have not been plowed in the last 100 years (pH 7.7) and supported an herb-rich plant community that was grazed. The contrasting high land use intensity treatment was cultivated as an arable cropland for cereal production, with a soil pH of 8 (Table 1). Pairwise *t*-tests were performed to ascertain the effect on land use intensity on soil properties.

**Table 1:** Site characteristics given as mean values (± standard error) with statistical comparison of land use intensity contrasts within sites given by *P* values of *t*-test.

	Low-pH site: Kirkton, Perthshire, UK			High-pH site: Parsonage Down, Wiltshire, UK		
Land use intensity	Low	High	Pairwise *t*-test *P* value	Low	High	Pairwise *t*-test *P* value
Land management	Unimproved grassland	Intensive grassland		Unimproved grassland	Intensive arable	
Soil pH	5.2 (±0.2)	6.4 (± 0.1)	.011	7.7 (± 0.03)	8.0 (± 0.04)	.001
Soil C (%)	23.8 (± 8.5)	4.3 (± 0.6)	.145	10.4 (± 0.5)	3.8 (± 0.1)	.004
Soil moisture (%)	72.1 (± 13.6)	41.7 (± 2.5)	.153	43.1 (± 4.9)	30.4 (± 4.9)	<.001

### Experimental design

Each site consisted of two land use intensity treatments resulting in four site-land use combinations. For each of these, three spatially dispersed soil cores (5 cm diameter, 15 cm deep) were sampled along a transect with 25 m between each core. Sampling was performed in February–March 2016. Soil samples were preserved at 4°C following removal of vegetation and homogenization by sieving (<4 mm). Mesocosms were established in Petri dish plates containing 10 g (dry weight equivalent) soil, maintained at field moisture gravimetrically and incubated at 21°C for 7 days. After this time, 3 mg ^13^C-labeled *Chenopodium* sp. leaf litter was mixed thoroughly with the soil in each mesocosm (*n* = 3). As the amount of carbon in the added litter was very low (<1%) relative to the existing soil carbon, the influence of litter addition on microbial community taxonomy and function is considered negligible. The ^13^C-labeled leaf litter was produced by growing *Chenopodium* sp. in a closed chamber containing ~1 atom% ^13^C-CO_2_ at a concentration of 400 ppm, followed by drying of leaves and homogenization by grinding. Mesocosms were destructively harvested on day 0 (just before litter addition) and days 2, 8, and 36 following litter addition. ^13^C-labeling of the litter enabled ^13^C to be traced into separate pools as microbial biomass and respired CO_2_. The labeled substrate was added at a single time, allowing the monitoring of the microbial channeling of C into biomass production and respiration over the incubation period, and inferring of ecosystem CUE estimates.

### Biomass production and respiration

An aliquot (1 g) of the soil collected at each sampling point was placed in a sealed 10-ml glass vial with rubber septa and incubated overnight (for ~16 h) at 21°C in the dark to collect respired CO_2_ in the headspace. Concentrations of CO_2_ and its ^13^C content was analyzed by gas chromatography isotope ratio mass spectrometer (GC-IRMS, Delta + XL, Thermo Fisher Scientific, Germany) coupled to a PAL autosampler (CTC Analytics) with general purpose interface (Thermo Fisher Scientific, Germany). Deoxyribonucleic acid (DNA) was extracted from 0.25 g soil at each sampling point using the PowerSoil-htp 96-well soil DNA isolation kit per manufacturer’s instructions (MO BIO Laboratories, UK) and its quality was checked by Nanodrop. Total extractable DNA concentration was also measured using a Qubit fluorometer, providing a proxy for microbial biomass [26]. ^13^C content of DNA extracts was analyzed by liquid chromatography isotope ratio mass spectrometer LC-IRMS (HPLC system coupled to a Delta + XP IRMS through an LC IsoLink interface; Thermo Fisher Scientific, Germany). This approach enabled quantification of the proportion of ^13^C-labeled plant litter in total microbial DNA and respired CO_2_ during the incubation. While ^13^C incorporation into DNA was a cumulative measure over the duration of experimental incubation, our respiration measurements were only performed for a duration of 16 h at each sampling point. This meant that we could not calculate ecosystem CUE, therefore we used the ratio of ^13^C incorporation into biomass and respiration to infer microbial ecosystem-level CUE. The distribution of residuals was checked for normality before performing statistical tests. Statistical analyses and visualizations in ggplot2 [39] were performed using R software 2023.3.0 [40]. Multi-factorial ANOVA was performed to ascertain the effect of site, land use intensity and sampling time on ^13^C in DNA, respired CO_2_, and its ratio.

### Metabarcoding

DNA was extracted as described above. Amplicon libraries were constructed according to a dual indexing strategy [41] with each primer consisting of the appropriate Illumina adapter, 8-nt index sequence, a 10-nt pad sequence, a 2-nt linker, and the amplicon specific primer. For prokaryotes, the V3-V4 16S ribosomal ribonucleic acid (rRNA) gene amplicon primers from Kozich et al. [41] were used (5′-CCTACGGGAGGCAGCAG-3′ and 5′-GCTATTGGAGCTGGAATTAC-3′); for eukaryotes the 18S rRNA gene amplicon primers from Baldwin et al. [42] were used (5′-AACCTGGTTGATCCTGCCAGT-3′ and 5′-GCTATTGGAGCTGGAATTAC-3′). Amplicons were generated using a high-fidelity DNA polymerase (Q5 Taq, New England Biolabs). After an initial denaturation at 95°C for 2 min polymerase chain reaction conditions were: denaturation at 95°C for 15 s; annealing at temperatures 55°C or 57°C (for 16S and 18S reactions, respectively); annealing times were 30 s with extension at 72°C for 30 s; cycle numbers were 25 for 16S and 30 for 18S; final extensions of 10 min at 72°C were included. Amplicon sizes were determined using an Agilent 2200 TapeStation system and libraries normalized using SequalPrep Normalization Plate Kit (Thermo Fisher Scientific) and quantified using Qubit dsDNA HS kit (Thermo Fisher Scientific). Each amplicon library was sequenced separately on Illumina MiSeq using V3 600 cycle reagents at concentrations of 8 pM with a 5% PhiX Illumina control library.

Sequenced paired-end reads were joined using PEAR [43] as per PIPITS [44], quality filtered using FASTX tools (hannonlab.cshl.edu), length filtered with the minimum length of 300 bp, presence of PhiX and adaptors were checked and removed with BBTools (jgi.doe.gov/data-and- tools/bbtools/), and chimeras were identified and removed with VSEARCH [45] using Greengenes 13_5 [46] and SILVA 132 [47] databases for 16S and 18S, respectively (at 97%). Singletons were removed and the resulting sequences were clustered into operational taxonomic units (OTUs) with VSEARCH at 97% sequence identity. Representative sequences for each OTU were taxonomically assigned by RDP Classifier [48] with the bootstrap threshold of 0.8 or greater using the Greengenes 13_5 and SILVA 132 databases (16S and 18S, respectively) as the reference (OTU tables in Supplementary data). Unless stated otherwise, default parameters were used for the steps listed. Taxonomic groupings of prokaryotes were presented using the older taxonomic classification to compare with proteomics-derived taxonomy. Only three major groups of eukaryotes: fungi, Ciliophora and Cercozoa were analyzed. α- diversity (Shannon Weiner diversity index) and β-diversity indices were calculated on rarefied data (to 14 746 reads for 16S, and 12 049 reads for 18 S) using the vegan package in R [49] and visualizations were performed using ggplot2 [39]. β-diversity was assessed by in non-metric multidimensional scaling ordinations and running permutational multivariate analysis of variance (PERMANOVA) using vegan’s adonis2 function. Multi-factorial ANOVA was performed to ascertain the effect of site and land use intensity on diversity indices and the abundance of taxonomic groups of interest.

### Metaproteomics

Metaproteomic analysis was performed on soil microbial communities for day 0 and day 8 samples. Proteins were extracted from 5 g of soil taken from each mesocosm (with two technical replicates) using the SDS buffer–phenol extraction method, followed by purification with 1D sodium dodecyl-sulfate polyacrylamide gel electrophoresis. The resultant product was subjected to tryptic digestion. Proteolytically cleaved peptides were separated prior to mass spectrometric analyses by reverse-phase nano-HPLC on a nano-HPLC system (Ultimate 3000 RSLC nano system, Thermo Fisher Scientific, San Jose, CA, USA) coupled online for analysis with a Q Exactive HF mass spectrometer (Thermo Fisher Scientific, San Jose, CA, USA) equipped with a nano electrospray ion source (Advion Triversa Nanomate, Ithaca, NY, USA). Raw data were searched using Proteome Discoverer v1.4.1.14 (Thermo Fisher Scientific) against a FASTA-formatted database (Uniprot 05/2016) using the SEQUEST HT algorithm. Additional details on quality control, database searches, and filtering are described elsewhere [26]. Functional annotation was performed using KEGG classifier and GhostKoala. Taxonomic origin was assigned to proteins using Unipept v3.2, enabling us to make function-taxonomy linkages. Data was normalized relative to total protein abundance and checked for normalized distribution. Two-factorial ANOVA was performed to ascertain the effect of site and land use intensity on proteomics-derived functional diversity index. Pairwise indicator species analysis was performed to identify the protein functions that were significantly enriched in low- and high-intensity land use treatments at each location [26]. The abundance of different protein functions that were identified was then investigated in each taxonomic group of interest and this was plotted using ggplot2 by combining the geom_tile and geom_point functions. Pairwise *t*-test was performed to ascertain the influence of distinct soil types and land use intensity on the abundance of protein functions associated with each taxonomic group.

## Results and discussion

### Land use intensification alters soil physicochemical properties

We compared local over-the-fence land use contrasts in two temperate sites of low- and high-soil pH to isolate the effect of land use intensification on soil properties while the climate and soil parent material remained constant. Land use intensification had profound effects on soil properties, significantly increasing soil pH at both sites (Table 1). At the low-pH Kirkton site, pH increased from 5.2 to 6.4 through liming that is performed to achieve the optimum soil pH range for crop plant nutrient availability [50]. Improved drainage and crop cultivation reduced the soil moisture that could reduce anoxia, further alleviating physiological constraints on the soil microbiome. Thus, the wider assumption that land use intensification causes aridity and drought stress [51] in soil microbiomes does not apply to poorly-drained acidic soils [26]. Land use intensification at the tested low-pH site resulted in >80% of the SOC being lost relative to the unimproved soil (Table 1). Increased decomposition in organic soils under land use intensification is a key mechanism for SOC loss, as the carbon at these sites is particularly vulnerable to loss due to a lower proportion of MAOM [4, 34, 52].

Land use intensification only marginally increased soil pH at the high-pH Parsonage Down site—a shift of 0.3 units. These soils are inherently alkaline, and do not require pH adjustment through liming to support agriculture. Increased land use intensification at this site reduced soil moisture, possibly increasing the risk of drought stress in these well-drained sites [53]. The effect of a single plowing event and conversion to cropland on previously uncultivated remnant prairie soil [54], comparable to the calcareous soil in our study, revealed little change in soil pH, but soil moisture was negatively impacted due to reduced water infiltration and sorptivity rates. Therefore, land use intensification likely creates drought like conditions at our high-pH site. Land use intensification at our high-pH site led to a marked SOC decline from 10.4% to 3.8% (Table 1), confirming that cultivated soils are prone to SOC loss [55]. This suggests that the observed increased soil pH under land use intensification led to reduced soil moisture availability and SOC loss at both sites but being most pronounced in low-pH soils.

### Land use intensification influences microbial growth but not respiration

**Figure 1:**
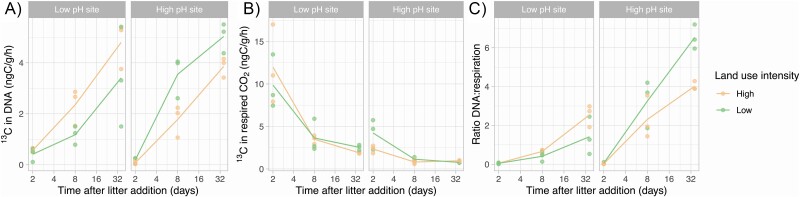
Microbial process rates across the paired land use intensity contrasts: isotopic incorporation from labeled litter into DNA as a measure of microbial biomass production (A) and into respired CO_2_ (B). Ratio of biomass production and respiration (C) provides an indication of ecosystem CUE. Points indicate individual samples, and the lines connect the mean values at each sampling time. ANOVA *P* values for (A) ^13^C in DNA – site: 0.37, treatment: 0.92, site × treatment: 0.006; (B) ^13^C in respired CO_2_ – site: <0.001, treatment: 0.95, site × treatment: 0.64; (C) ratio of biomass production and respiration – site: <0.001, treatment: 0.6, site × treatment: <0.001.

Microbial growth measured as ^13^C substrate incorporation into DNA (Fig. 1A), increased with land use intensification at the low-pH site (54% more in the high than in the low land use intensity soil) and decreased at the high-pH site (35% less in the high than in the low land use intensity soil). This contrasting effect of land use intensity at the two sites is corroborated by the significant interactive effect of site and land use intensity (ANOVA, *P* < 0.001). This result supports our hypothesis that land use intensification reduces carbon incorporation into microbial biomass, but only at the high-pH site where land use intensification reduced soil resource availability and moisture. In contrast, land use intensification at low-pH alleviated physiological constraints of acidity, wetness, and anoxia, enabling increased growth. We hypothesized that land use intensification results in an increase in the decomposition rate of an added complex resource, measured by an increased ^13^CO_2_ production. However, there was no difference in respiratory rate of the ^13^C-labeled substrates in soils across the land use intensity treatments at both sites (Fig. 1B).

The ratio of ^13^C in microbial DNA and respired CO_2_ as an estimate of microbial ecosystem-level CUE was not statistically significant across land use intensity treatments, but the interaction of site and land use intensity treatment was significant (ANOVA, *P* < .001). This pattern suggests that there was a reduction in the inferred CUE with land use intensification at the high-pH site and the opposite at the low-pH site (Fig. 1C). The increased biomass production values over time following labeled litter addition highlights the long-term persistence of carbon in the microbial biomass due to substrate recycling in the microbial food web. Such measurements are key to studying the longer-term effects of microbial processes on soil carbon cycling; measurements over a longer incubation period (several weeks) enables inferring the complex interactions within the microbial community and between the microbial community and its abiotic environment [9]. The reduction in biomass production with land use intensification at the high-pH site translates into lower biomass and necromass production with a lower SOC stabilization potential [13, 56]. Conversely, land use intensification alleviated environmental stressors on the soil microbiome in low-pH soil, promoting microbial growth. Here SOC change is decoupled from microbial production, and other biogeochemical mechanisms might be more important in controlling the rate of SOC loss or accumulation. It also highlights that current microbial CUE measurements do not always link to historical soil carbon changes. Therefore, future research must consider the balance between the biogeochemical processes of decomposition and stabilization, including abiotic factors such as organic matter access, chemistry, and mineral stabilization, when studying the impact of long-term land use change on changes in soil carbon storage.

### Land use intensification changes microbial diversity

**Figure 2:**
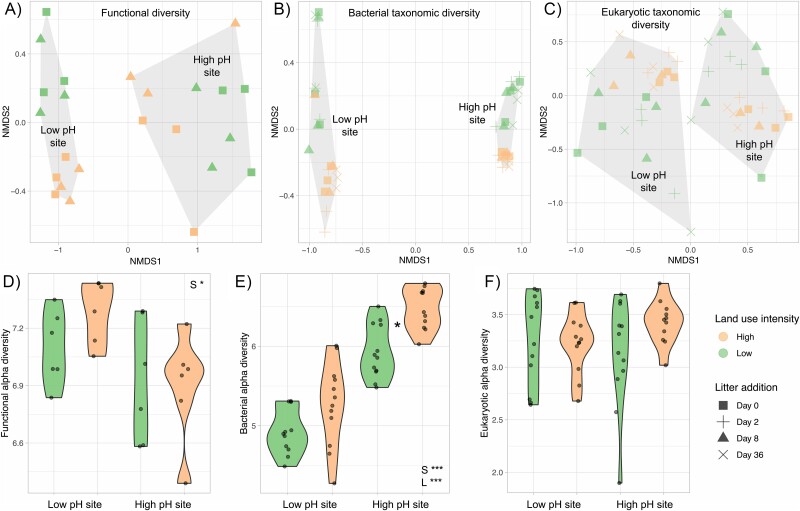
Microbial functional and taxonomic diversity across the paired land use intensity contrasts: ordination using nonmetric multidimensional scaling based on Bray–Curtis dissimilarity of (A) metaproteomics-derived functions, stress = 0.06, PERMANOVA results – site: *P* = .12, land use intensity: *P* = .64; (B) 16S rRNA gene-derived bacterial taxonomy, stress = 0.30, PERMANOVA results – site: *P* = .20; land use intensity: *P* = .04, and (C) 18S rRNA gene-derived eukaryotic taxonomy, stress = 0.03, PERMANOVA results – site F = 1.35, *P* = .17; land use intensity: *P* = .07. Similarly, Shannon’s diversity index was used to visualize functional alpha diversity (D), bacterial alpha diversity (E), and eukaryotic alpha diversity (F) under high and low land use intensity at the two sites under study. In D–F, the presence of an asterisk between low and high land use intensity violins suggests statistically significant pairwise differences from Tukey’s HSD test. Also displayed within D–F are statistically significant ANOVA results of the influencing factors of site (S), land use intensity (L) and their interaction (S × L); ^*^^*^^*^P < .001, ^*^^*^P < .01, ^*^P < .05 (non-significant results are not displayed). Note that metaproteomics was performed only at day 0 and day 8 after litter addition.

The functional (inferred from metaproteomics) and taxonomic (inferred from metabarcoding) composition of microbial communities appeared distinct for land use intensity treatments in the two sites based on the separation of samples in an ordination (Fig. 2A–C) but was only significant for bacterial taxonomic diversity (PERMANOVA *P* = .04). Functional and eukaryotic taxonomic alpha diversity was not different across the land use intensity treatments at both sites (Fig. 2D, ANOVA *P* > .05), but bacterial taxonomic alpha diversity was higher at higher land use intensity treatment (ANOVA *P* < .001). The community shifts over time were insignificant, suggesting that the small amount of plant litter that was added caused only minor changes in microbial taxonomy and function; all sampling points were therefore considered replicates to study the effect of land use intensification.

**Figure 3:**
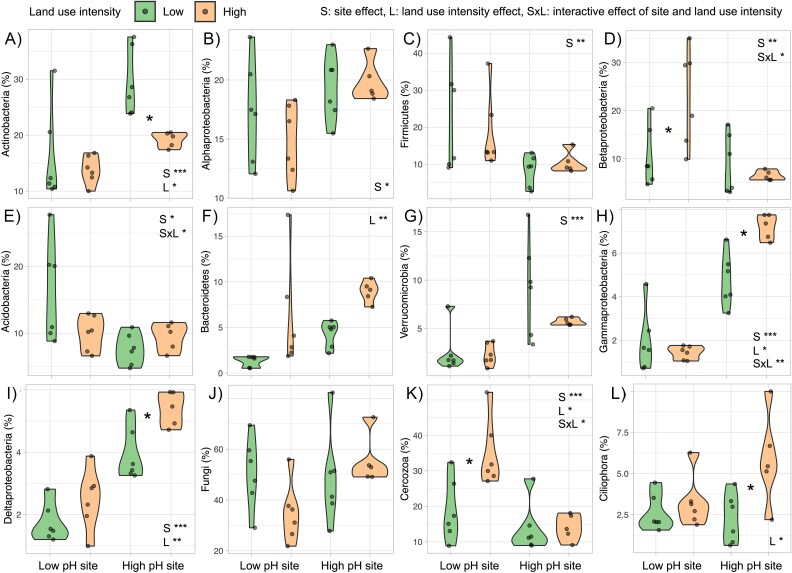
Microbial compositional differences in low and high land use intensity treatments: relative abundance of dominant bacterial and eukaryotic phyla/class: Actinobacteria (A), Alphaproteobacteria (B), Firmicutes (C), Betaproteobacteria (D), Acidobacteria (E), Bacteroidetes (F), Verrucomicrobia (G), Gammaproteobacteria (H), Deltaproteobacteria (I), Fungi (J), Cercozoa (K), and Ciliophora (L). Abundances are displayed across land use intensity treatments and the presence of an asterisk between the violins suggests statistically significant pairwise differences at the site from Tukey’s HSD test. Also displayed within each plot are statistically significant ANOVA results of the influencing factors of site (S), land use intensity (L) and their interaction (S × L); ^*^^*^^*^P < .001, ^*^^*^P < .01, ^*^P < .05.

Higher bacterial alpha diversity with land use intensification (Fig. 2E) corroborates previously observed high bacterial diversity in agricultural soils [29, 56], contradicting the notion that disturbance decreases biodiversity [57]. Several explanations for this apparent paradox have been proposed, such as agricultural rotations increasing resource heterogeneity [58] and tillage redistributing plant litter to depth facilitating access to resources and growth of a diverse range of bacteria [59]. The high diversity of microbial taxa in agricultural soils could also represent relic DNA from dead microbes that sticks to soil minerals [60]. In contrast, eukaryotic alpha diversity (Fig. 2F) and the abundance of OTUs representing fungal taxa (Fig. 3J) were unaffected by land use intensification.

The relative abundance of the 12 most abundant taxa (bacteria, fungi, and microeukaryotes) were differentially affected by the sites and land use intensity. At the high-pH site, low land use intensity soil bacteria were dominated by Actinobacteria, Alphaproteobacteria, and Verrucomicrobia (Fig. 3A, B, and G). Land use intensification in high-pH soil reduced the relative abundance of Actinobacteria, but increased that of Gammaproteobacteria, Deltaproteobacteria, and Ciliophora (Fig. 3H, I, and L). The decline of Actinobacteria under high land use intensity accords with Griffiths et al. [27] who noted that Actinobacteria are common in higher pH soils, but being filamentous, are sensitive to disturbances from agricultural management [61, 62]. Acidobacteria was one of the most dominant bacterial groups in low intensity soils at the low-pH site (Fig. 3E). Land use intensification at this site increased the relative abundance of Betaproteobacteria (from 9% to 26%, Fig. 3D), which has been observed in previous studies [63]. Land use intensification at the low-pH site also significantly increased the relative abundance of predatory Cercozoa (from 15% to 23%, Fig. 3K). While directly comparing relative abundances of different taxa across treatments can sometimes be misleading, here we compared metabarcoding-derived relative abundance with abundance of protein functions across taxonomic groups. We believe that such cross comparison validates our results obtained from the two complementary tools.

### Taxa-trait changes due to land use intensification in the low-pH site

**Figure 4:**
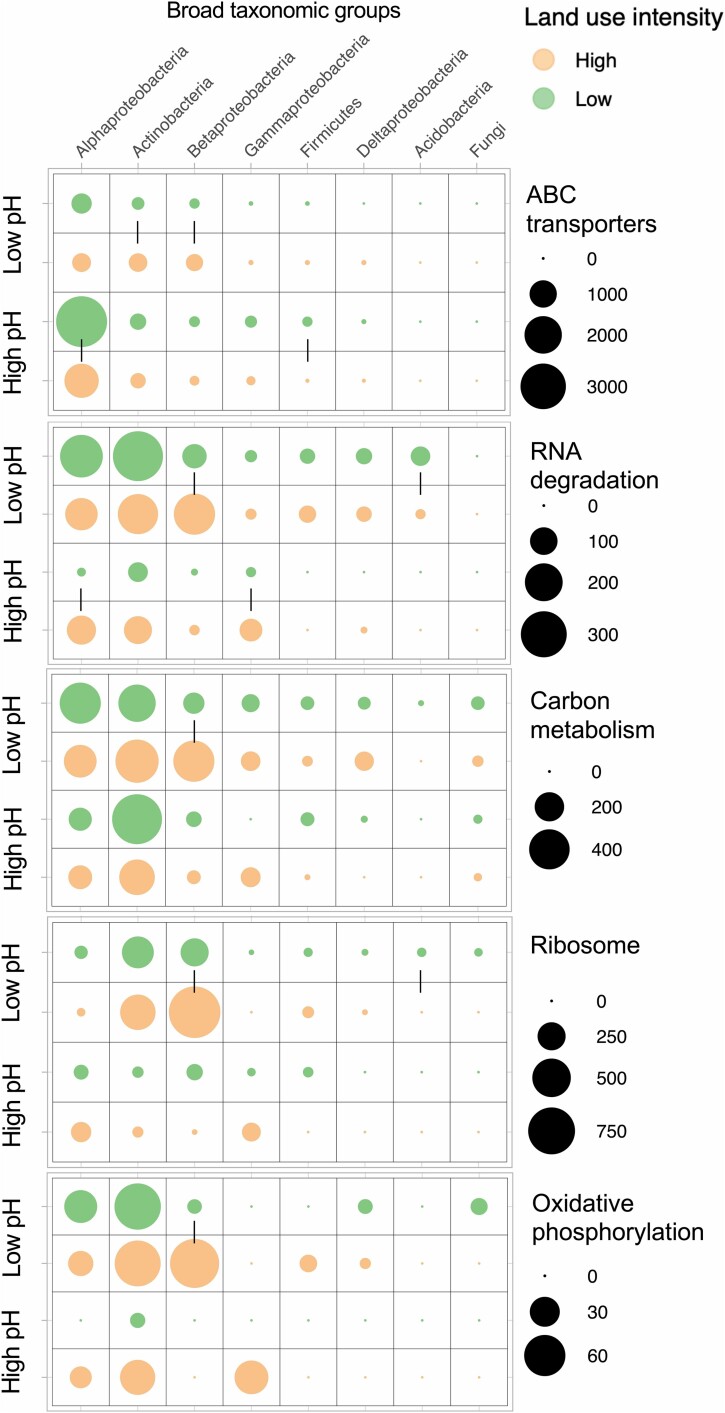
Trade-offs in traits across taxonomic groups in land use intensity treatments: Metaproteomics-derived abundances of functions and their taxonomic lineages were used to link physiological traits to microbial taxa at high and low land use intensity at the two sites (low- and high-pH). Pairs of circles representing peptide abundances linked by a vertical line within each site are significantly different (*P* < .05).

“RNA degradation” proteins were the most abundant protein indicators at the low-pH site in both low and high land use intensity treatments but with higher relative abundances in the low intensity land use treatment (Fig. 4). RNA degradation proteins such as Chaperonin GroEL and molecular chaperone DnaK prevent protein aggregation by either re-folding or degrading stress-induced misfolded proteins [30]. Chaperone production in high land use intensity soils indicates microbial investment into stress tolerance. This is likely a physiological response to the acidic and wet conditions in the low land use intensity soils [26]. They were differentially abundant in the phylum Acidobacteria in the low land use intensity relative to high land use intensity soils (Acidobacteria was one of the most dominant taxonomic groups in low land use intensity soils at the low-pH site). There were other taxa that also had higher expression of this trait in the low land use intensity soils.

Land use intensification at the low-pH site increased the abundance of Betaproteobacteria which showed differentially abundant stress proteins (“RNA degradation”) in high land use intensity soils. However, they also showed increased abundance of “ABC transporters” (Fig. 4) associated with a transporter-mediated resource acquisition strategy that is likely more efficient in resource use [26]. These communities with an uptake-optimized resource acquisition strategy reflect the increased abundance of resources under high land use intensity treatment. This is a result of alleviation of constraints on microbial organic matter decomposition due to increase in pH and decrease in wetness and anoxia. Betaproteobacteria also had increased expression of proteins linked to “carbon metabolism”, “ribosome”, and “oxidative phosphorylation” pathways; a land use intensification response very similar to that of Gammaproteobacteria in high-pH soils. “Oxidative phosphorylation” proteins represent energy generating pathways using ATPase to fuel growth or non-growth maintenance activities, and “carbon metabolism” proteins represent central carbon metabolism pathways such glycolysis and TCA cycle [26]. This response by Betaproteobacteria likely represents a shift toward increased growth and turnover in a stressed and disturbed environment. Land use intensification also significantly increased the relative abundance of predatory Cercozoa (from 15% to 23%, Fig. 3K) at low-pH, which may be responding to increased prey availability under high intensity land use, such as the increase in fast-growing Betaproteobacteria. The increase of Cercozoa under high land use intensity at the low-pH site mirrors the increase of Ciliophora under high land use intensity at the high-pH site, which suggests that the dominance of distinct bacterial groups might be associated with distinct predatory protozoan groups driving turnover of carbon to a variable degree.

### Taxa-trait changes due to land use intensification in the high-pH site

“ABC transporters” were the most abundant protein indicators of low intensity land use at the high-pH site reflecting abundant high-quality resource availability most likely as root exudates and microbial-derived metabolites. The taxonomic assignment of these transporters suggested that they were mostly associated with Alphaproteobacteria (Fig. 4). Although Alphaproteobacteria were not differentially abundant in low land use intensity soils compared to the high land use contrast at this site, in terms of the taxonomic distribution of this trait, Alphaproteobacteria were the dominant class differentially expressing this function in low land use soils. This implies that members of this class have a resource-uptake optimized strategy in low land use intensity soils that could contribute to increased community-level CUE, which could therefore promote SOC stabilization [30].

We observed that land use intensification in high-pH soils increased the expression of proteins linked to “RNA degradation”, indicating stress tolerance. This trait was differentially expressed in the taxa Alphaproteobacteria and Gammaproteobacteria (Fig. 4). Members of these taxa in high land use intensity soils likely excel in a stress tolerance strategy to tide over the dry and disturbed soil conditions.

In addition to the increased expression of stress tolerance traits in Gammaproteobacteria in high land use intensity, proteins linked to “oxidative phosphorylation” and “carbon metabolism” were abundant in high land use intensity soils but were not detected in low land use intensity (Fig. 4). This most likely represents increased energy needs for fast growing taxa with a wasteful metabolism; a life history strategy often associated with copiotrophs such as Gammaproteobacteria that are differentially abundant in high land use intensity soils at this site [26]. We also observed concomitant increased abundance of predatory Ciliophora in high land use intensity soils, these likely increase in response to the increased abundance of their bacterial prey—a hypothesis that needs testing [64]. These microbivorous protists could contribute to SOC stabilization directly through increased necromass contributions, but also through their influence on the assemblage and function of the microbiome [64].

### Taxa-trait changes related to mechanisms of soil carbon cycling

The observed shifts in trait-taxa linkages are in line with our hypothesis that land use intensification leads to shifts in microbiome composition and its associated traits that has consequences for soil carbon cycling. In our high-pH site, low intensity land use with no resource limitation and minimum stress resulted in a microbial community that is dominated by taxonomic sub-groups within Alphaproteobacteria that have an efficient transporter-mediated resource-uptake optimized life history strategy with limited investment in stress tolerance traits. This likely increased the microbial biomass (and therefore necromass production) promoting SOC stabilization pathways. However, increased land use intensification in high-pH soils caused resource limitation and stress in microbes which lead to proliferation of microbial sub-groups within Alphaproteobacteria and Gammaproteobacteria that likely excel in an inefficient stress-tolerance life history strategy diverting resources away from biosynthesis and necromass formation and resulted in increased carbon loss and reduced SOC stabilization. The taxa-trait linkages were vastly different in low-pH soils. Here, soils under low intensity land use were dominated by Acidobacteria excelling in stress tolerance traits highlighting a life history strategy that is adapted to the acidic, wet, and anoxic soil conditions. The low growth rates observed in these soils suggest lower rates of decomposition and accumulation of undecomposed plant organic matter. Increased land use intensification in these low-pH soils reduced soil acidity, wetness and anoxia which led to increased microbial growth likely due to alleviation of microbial physiological constraints. This results in a shift toward Betaproteobacteria excelling in stress tolerance and resource acquisition strategies that fuel their higher growth rates which could be linked to increased decomposition and loss of the historically accumulated SOC.

Our research reveals that land use intensification induced shifts in the microbial taxa and their life history strategies were pH-dependent, and changes in soil characteristics selects for a new community with different traits (environmental filtering) rather than the community shifting its physiology (phenotypic modification) [4, 33]. Our study accords with previous trait-based approaches that have demonstrated that microbial efficiency declines along gradients of environmental stress, as increased stress through altitude [35] and salinity [37] results in increased stress tolerance and resource acquisition life history strategies that reduce microbial CUE and negatively influence the microbially-derived SOC formation. Further, our findings of increased abundance of predatory protozoa in response to increased land use intensification, could be crucial for carbon turnover and food web connectivity. This is especially pertinent, as protists are known to be key for promoting the formation of necromass and consequently more persistent MAOM [62].

Here, we successfully used a trait-based framework to link taxonomic information to traits and rates of carbon cycling in soils. In this sense, this approach encompasses many of the concepts required to envisage soil health [65] by focusing on the function of the active microbiome and its emergent traits but also on other biogeochemical factors that are key to determining the balance of SOC decomposition and stabilization pathways. We also demonstrate how CUE–SOC relationship can be decoupled and how variable pathways of decomposition and stabilization of POM and MAOM can influence SOC loss or gain in response to land use change. This holistic understanding will be fundamental to predict soil’s ability to recover from the combined stressors of intensification along with environmental change, to ensure that our soils and their resident microbiomes remain resilient and productive under global change [66].

## Supplementary Material

otu_table_16s_Table_S1_ycae116

otu_table_18s_Table_S2_ycae116

## Data Availability

The metabarcoding datasets generated during the current study are available in NCBI SRA repository (https://www.ncbi.nlm.nih.gov/sra/PRJNA1088078). Annotated OTU data are included in this published article (Supplementary information files S1 and S2). The proteomics mass spectrometry data generated during the current study are available in the ProteomeXchange Consortium via the PRIDE partner repository (https://www.ebi.ac.uk/pride/archive/projects/PXD010526).
